# Green Synthesis of a Hyaluronan-Based Multimeric A9 Peptide System: Polysaccharide-A9 Conjugate with Enhanced HER2 Receptor Binding Affinity and Potential Biomedical Applications as an Active Material

**DOI:** 10.1021/acs.biomac.5c01553

**Published:** 2025-11-18

**Authors:** Valentina Verdoliva, Alfio Pulvirenti, Giuseppe Digilio, Stefania De Luca

**Affiliations:** † Department of Environmental, Biological and Pharmaceutical Sciences and Technologies, National Research Council (CNR), 296400 Institute of Crystallography, Via Vivaldi, 43, 81100 Caserta, Italy; ‡ Department of Biomedical Sciences, 366187 Institute of Biostructures and Bioimaging, National Research Council (CNR), Via P. Castellino, 111, 80131 Naples, Italy; § Department of Science and Technological Innovation, 19050 Università del Piemonte Orientale “A. Avogadro”, 15121 Alessandria, Italy

## Abstract

A solid-state strategy for conjugating the peptide sequence A9 to hyaluronic acid (HA) has been developed via mechanosynthesis promoted by microwave radiation. The method employs a silica-supported nucleophilic catalyst (DMAP) to activate the carboxylic groups of the polysaccharide, enabling efficient conjugation. These conditions minimize waste generation, enhancing environmental sustainability and operator safety while ensuring a high yield of the final HA-A9 conjugate. The multicopy HA-A9 system exhibits enhanced affinity toward a synthetic HER2 receptor model. Furthermore, the HA-A9 conjugate demonstrates the ability to self-assemble into micellar aggregates and form viscous materials enriched with β-sheet peptide structures. These supramolecular assemblies highlight the potential of conjugates for biomedical applications.

## Graphical abstract

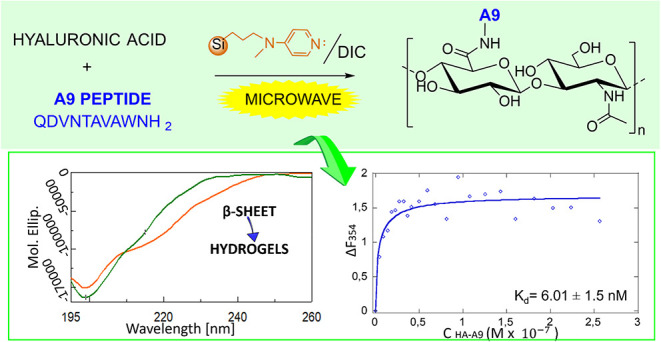

## Introduction

Recently, we have explored several reliable, effective, time-saving, and environmentally friendly methods for functionalizing natural polysaccharides (pectin and hyaluronic acid) with rather hydrophobic compounds (natural fatty acid, curcumin, and quercetin).^1–4^ These approaches posed a significant challenge in synthetic chemistry involving partial or complete solvent-free procedures to conjugate two molecules with different solubility profiles. A mechanochemical method, utilizing ball milling operation, was employed to carry out the chemical transformations without the use of a solvent.^5–10^ This approach provides a more sustainable and efficient alternative to the traditional solution-based methods. It is worth noting that this technique provides the energy necessary for the collision of reactants, thereby promoting the reaction. Moreover, the implementation of alternative energy sources, such as microwave radiation (MW), was proven to be highly effective, particularly for reaction mixtures containing one or two polar or ionic components.^4,11,12^

The A9 peptide (H_2_N–QDVNTAVAW–CONH_2_) is a nine-residue linear sequence that binds with high affinity to the HER2 receptor, making it a promising candidate for HER2 inhibition.^13–17^ However, its pronounced hydrophobicity limits solubility in physiological media and contributes to nonspecific binding through interactions with hydrophobic biological structures in tissues. Recently, we reported a microwave-enhanced mechanochemical approach for linking two A9 units via a PEG linker, resulting in a dimer with improved drug properties, including increased solubility and enhanced binding affinity to its molecular target.^14^ Motivated by the appealing advantages of mechanochemistry and the encouraging outcomes of A9 dimerization, we envisioned that this protocol could be extended to conjugate several copies of the A9 peptide to natural polysaccharides, such as hyaluronic acid (HA).

Functionalizing natural polysaccharides such as HA with multiple copies of the same peptide is a valuable strategy to enhance and diversify their biomedical applications. In fact, this approach can modulate properties, such as bioactivity, cell interaction, and scaffold stability. Hyaluronic acid, a naturally occurring, hydrophilic polysaccharide prevalent in the extracellular matrix (ECM), is particularly attractive for such modifications due to its biodegradability and intrinsic capacity to interact with various cell types. Conjugation of HA with bioactive peptides has been explored extensively.^18–20^

Here, we present a solvent-free synthetic method based on solid-state, mechanically assisted conjugation. The reaction was driven by microwave radiation, which provided the requested energy. The activation of the carboxylic groups on the polysaccharide was carried out with a solid-supported organic base, specifically 4-(dimethylamino)­pyridine (DMAP).^21,22^ The next step involved a conjugation reaction with the amino-terminal group of a fully deprotected A9 peptide sequence. A relevant aspect of this approach is that the organic base can be removed simply by filtration and the solid-supported reagent can be reused. This makes the entire conjugation process more environmentally friendly, reducing the level of chemical waste and consumption. A comprehensive structural characterization of the synthesized amphiphilic conjugate was performed using UV–vis, FT-IR and NMR spectroscopy. Additionally, the binding affinity of the conjugate toward the HER2-DIVMP receptor model was evaluated through a fluorescence spectroscopic method, which enables a thorough assessment of the A9 interaction properties and its potential biological activity.

## Experimental Section

### Materials

Fmoc protected amino acids, Rink Amide MBHA resin, *N*-hydroxybenzotriazole (HOBt) and benzotriazol-1-yl-oxy-tris-pyrrolidino-phosphonium (PyBOP), HA sodium salt from *Streptococcus equi* (15–30 kDa molecular weight), Spectra-Por Float-A-Lyzer G2 dialysis device (MWCO 6–8 kDa), and all solvents were purchased from Sigma (Sigma-Aldrich, St. Louis, MO, USA); piperidine and diisopropylethylamine (DIPEA) were purchased from Fluka (Milwaukee, WI, USA); SiliaBond DMAP was purchased from Silicycle (Québec, QC, G1P 4S6, Canada)

### Synthetic Procedures and Characterization of HA-A9 Conjugates

#### Synthesis of A9

Peptide sequences were obtained amidated at their C-termini by employing a Rink Amide MBHA resin (0.74 mmol g^–1^ substitution; 50 μmol scale). The coupling protocol uses oxyma and DIC (oxyma/DIC) as activating reagents

Standard Fmoc-protecting group strategy, performed on solid phase, was employed by the automated Peptide Synthesizer Syro I from Multi-SynTech GmbH (Witten, Germany). The instrument is equipped with a One U-Type Reactor Block featuring 24 positions and 5 mL reactors (PP-reactors, 5 mL with TF frit, Cod. V050TF062, MultiSynTech GmbH, Witten, Germany).

Additionally, mixing of the coupling reactions was obtained through a vortex included in the Syro I system. Fmoc deprotection was carried out with 20% piperidine in DMF for 5 + 10 min. When necessary, amino acid coupling steps were monitored by manually performing the Kaiser test after coupling cycles.

The obtained protected peptide-resins were treated with a highly concentrated acidic solution (TFA/H_2_O/TIS; 97:2:1) for an average time of 3 h. This operation allowed the concomitant cleavage from the solid support and simultaneous removal of all protecting groups from the amino acid side chains. Then, the first purification step consisted of a precipitation operation, performed in cold diethyl ether. This allowed the isolation of the peptide, subsequently collected as a white solid after centrifugation.

All RP-HPLC chromatographic procedures employed H_2_O with 0.1% TFA (A) and CH_3_CN with 0.1% TFA (B) as a solvent system. The detection was carried out at 210 and 280 nm.

HP Agilent Series 1200 apparatus equipped with a Phenomenex (Torrance, California) Gemini column (5 μm NX-C18 110 Å, 150 × 21.2 mm^2^, AXIATM), with a flow rate of 15 mL min^–1^ and a linear gradient ranging from 5% to 70% B over 20 min, was employed as a preparative purification instrument.

Identification was performed by LC-ESI-MS analyses with an Agilent 1260 Infinity II system coupled to an LC/MSD XT single quadrupole mass spectrometer (Agilent Technologies, Cernusco sul Naviglio, Italy) and a Phenomenex (Torrance, California) Jupiter column (3 μm C18 300 Å, 150 × 2.0 mm^2^). The solvent system was H_2_O with 0.05% TFA (A) and CH_3_CN with 0.05% TFA (B). A linear gradient from 5% to 70% B over 20 min was run.

#### Synthesis of HA-A9 Conjugates

Ten mg of HA and 10 μL of *N,N′*-diisopropylcarbodiimide (DIC,0.0060 mmol) were manually ground in an agate mortar in the presence of silica-supported 4-(dimethylamino)­pyridine (SiliaBond DMAP, 0.012 mmol, substitution degree: 0.92 mmol g^–1^). The resulting mixture was transferred to a 0.5–2.0 mL microwave vial and irradiated at 80 °C for 2 min using a Biotage Initiator+ microwave reactor (Sweden AB, Uppsala, Sweden).

In a subsequent step, the activated HA was manually ground with 5 mg of A9 (0.0050 mmol) in the presence of potassium carbonate (K_2_CO_3_, ∼3 mg, catalytic amount) using an agate mortar. The reaction mixture was allowed to cool to room temperature and then dissolved in Milli-Q water (∼20 mL) and filtered through paper to remove the silica.

In this step, the insoluble byproduct *N*,*N*′-diisopropylurea (DCU) was efficiently removed by filtration together with the silica-supported DMAP.

The aqueous solution was dialyzed (Spectra-Por Float-A-Lyzer G2, MWCO 6–8 kDa) against Milli-Q water for 24 h. The obtained solution was lyophilized to isolate the HA-A9 conjugate as a solid product.

#### FT-IR Spectroscopy Characterization

HA-A9 conjugates were characterized by FT-IR spectroscopy using the ATR accessory of the JASCO FT/IR-4100 Fourier Transform Infrared Spectrometer instrument. IR transmission spectra were recorded with a number of scans of 16 at a resolution of 4 cm^–1^ over a wavenumber region of 400–4000 cm^–1^. The relevant bands of the HA-A9 conjugates are 3407 cm^–1^ (alcoholic O–H stretching), 3277 cm^–1^ (N–H amine II stretching), 2930 cm^–1^ (C–H stretching), 1663 cm^–1^ (N–H amide I), 1624 cm^–1^ (deprotonated carboxyl C=O stretching), 1532 cm^–1^ (N–H amide II bending), 1410 cm^–1^ (COO^–^ bending), and 1078 and 1046 cm^–1^ (C–O–C) glycosidic bond ring.

#### UV–vis Spectroscopy Characterization

A9 concentration in the HA-A9 conjugate was evaluated by absorbance measurements (λ_max_ = 280 nm; ε = 5630 M^–1^ cm^–1^) using a Jasco V-730 Spectrophotometer_ETCS-761.

UV–Vis range chosen for the recorded spectra was 250–600 nm. Quartz cells of 500 μL were employed to perform the measurements at room temperature. A blank spectrum was recorded to correct for all collected spectra. The parameter settings were as follows: scan speed of 200 nm/min, data interval of 0.2 nm, response time of 0.24 s, continuous scan mode, and a bandwidth of 1.0 nm.

#### Fluorescence Spectroscopy Characterization

JASCO FP-8350 ETC-115 spectrofluorometer equipped with a 1.0 cm quartz cuvette was employed to record fluorescence emission spectra of the HA–A9 conjugate (0.046 μM) at room temperature using a. The excitation wavelength was set at 280 nm, and emission spectra were recorded in the range of 300–550 nm. Other instrumental parameter settings are as follows: scan speed of 200 nm/min; data interval of 0.5 nm; and sensitivity, medium.

In a typical titration experiment, 1 mL of the HER2-DIVMP solution (0.1496 μM) in 10 mM phosphate buffer (pH = 7.2) was titrated with aliquots of HA-A9 stock solution (0.256 μM) prepared in the same buffer. After each addition, the samples were mixed and allowed to equilibrate before fluorescence measurements.

To estimate the fluorescence changes due to the interaction between HA-A9 and HER2-DIVMP, a blank titration was performed by adding the peptide aliquots to the same volume of the buffer solution. Final spectra were corrected for blank and adjusted for dilution, and the individual fluorescence contributions of the peptide and receptor fragment were subtracted from the total registered signal.

All titrations were performed in triplicate. Fluorescence intensity at 354 nm was plotted versus A9 peptide concentration and fitted using a sigmoidal dose–response binding model in KaleidaGraph.

#### Circular Dichroism Characterization

HA-A9 conjugates were analyzed by circular dichroism (CD) spectroscopy using a Jasco J-1500 CD spectrometer.

Measurements were performed at 20 °C in a quartz cuvette with a path length of 0.1 cm. The conjugates were studied at two different concentrations, 0.026 mM (dark green) and 0.13 mM (orange) (Figure 7), and at two different pH values (pH = 1 and pH = 7). The spectra were normalized to the mean residue ellipticity ([θ]), and the secondary structure content was estimated accordingly.

#### Preparation and Size Distribution Characterization of HA-A9 Micelles (Dynamic Light Scattering)

HA-A9 nanoparticles were prepared by a sonication method. Briefly, 1.0 mg of the HA-A9 conjugate was dissolved in 1 mL of 0.9% (w/v) NaCl aqueous solution. The solution was sonicated for 30 min at room temperature to facilitate nanoparticle formation. Subsequently, the suspension was centrifuged at 13,000 rpm for 10 min.

Nanoparticle size and distribution were characterized by dynamic light scattering (DLS) using a Zetasizer PRO (Malvern Panalytical, Worcestershire, United Kingdom; Almelo, Netherlands). Measurements were performed at 25.0 °C in disposable microcuvettes with a volume capacity of 40–45 μL.

#### Determination of Critical Micellar Concentration

A stock solution of pyrene (3.0 × 10^–2^ M in ethanol) was diluted with Milli-Q water to achieve a final pyrene concentration of 1.2 × 10^–7^ M. Ethanol was removed using a rotary evaporator at 60 °C for 1 h, yielding an aqueous pyrene solution with a final concentration of 4.8 × 10^–7^ M.

The HA-A9 conjugate was dissolved in the aqueous pyrene solution (4.8 × 10^–7^ M) to obtain a series of concentrations: 1.7, 1.25, 0.62, 0.31, 0.16, 0.08, 0.04, 0.02, 0.01, 0.005, 0.002, 0.0012, 0.0006, 0.0003, 0.00015 mg mL^–1^.

Fluorescence emission spectra were recorded from 350 to 500 nm by using a JASCO FP-8350 ETC-115 spectrofluorometer with an excitation wavelength of 336 nm. The intensity ratio of the first (I_1_, 373 nm) to the third (I_3_, 384 nm) vibronic peaks of pyrene was plotted against the concentration of the HA-A9 conjugate.

CMC is set as the intersection of the two straight lines presumably corresponding to the premicellar and the postmicellar regions.

#### Nuclear Magnetic Resonance (^1^H NMR) characterization

NMR spectra were acquired with a Bruker Avance spectrometer operating at 14 T (corresponding to a proton Larmor frequency of 600 MHz), equipped with an inverse Z-gradient 5 mm BBI probe. A9-HA was dissolved in a mixture of H_2_O (600 μL) and D_2_O (50 μL) to a concentration of around 0.2 mg/mL. If necessary, the pH was adjusted between 5 and 6. ^1^H NMR spectra were acquired at 298 ± 0.1 K by means of a pulse sequence with water suppression using excitation sculpting with gradients (Bruker pulseprogram zgesgp). Acquisition parameters were as follows: relaxation delay 2.5 s, spectral width 16 ppm, time domain complex data points 32,768, and number of scans 2048. This acquisition scheme allowed us to detect A9 peptide amide resonances without possible artifacts, possibly due to saturation transfer. To measure the degree of substitution, the Bruker pulse program noesy1dgppr was used, with presaturation during the relaxation delay and mixing time and with a spoil gradient for water suppression. Acquisition parameters included a relaxation delay of 4 s and a minimum number of scans of 256. The methyl signal of the *N*-Acetyl-glucosamine moiety of HA and the valine methyl signals at 0.81 ppm of A9 were integrated and used to calculate the degree of substitution (DS)

$$\mathrm{DS}=\frac{n_{A9}}{n_{\mathrm{RU}}}=\frac{1}{2}\times \frac{I_{\mathrm{Val}-A9}}{I_{\mathrm{Ac}-\mathrm{RU}}}$$

where *n*_A9_ are the moles of A9 bound to HA, *n*_RU_ are the moles of repetitive units (RU) in HA, *I*_Val‑A9_ is the integral of the A9 valine signal at 0.8 ppm, and *I*_Ac‑RU_ is the integral of the HA acetate peak at 2.0 ppm.

## Results and Discussion

### Synthesis

Multivalent ligand systems are developed to simultaneously engage multiple target binding sites. The final aim is to enhance properties, such as binding affinity, specificity, and, ultimately, to improve biological activity.^14,15^

Therefore, we developed a solvent-free synthetic strategy to conjugate multiple copies of the A9 peptide sequence to hyaluronic acid (HA) (Scheme 1). Since the hydrophilicity of HA and the hydrophobic nature of the A9 peptide pose significant solubility challenges for traditional solution-based methods, performing the reaction in the absence of solvent allowed us to overcome solubility issues. The synthesis involved amidation of hyaluronan carboxylic groups with free N-terminal amine of A9.

**Scheme 1:**
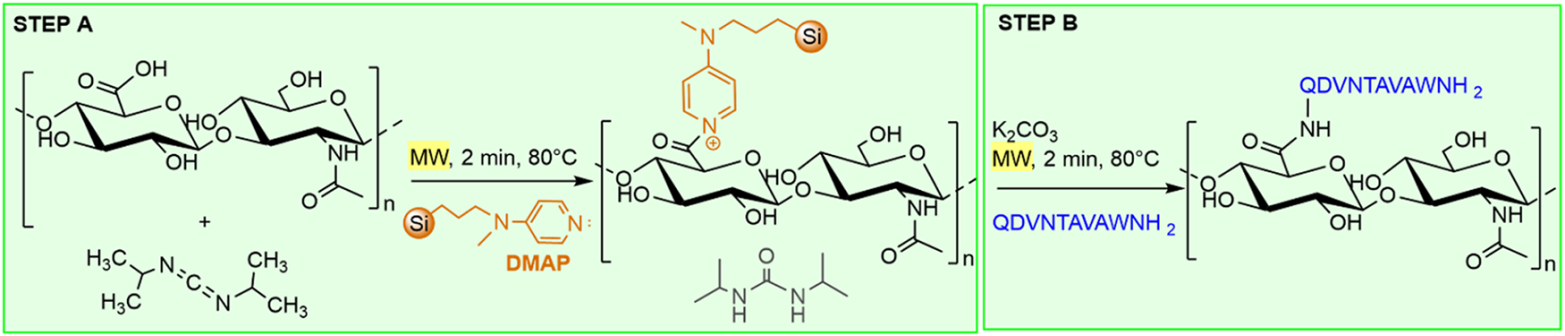
Solid-State Synthetic Strategy of HA-A9 Conjugates

Following a previously established protocol, the activation of the hyaluronan carboxylic groups was achieved by using *N*,*N*′-diisopropylcarbodiimide (DIC). The reaction was conducted under solvent-free conditions and employed 4-dimethylaminopyridine (DMAP) supported on silica as the base (Steglich esterification conditions).

DMAP is an inexpensive and widely used nucleophilic and basic organo-catalyst that has demonstrated high efficiency across numerous organic transformations. However, a significant toxicity is associated with residual DMAP, and this restricts its use. To mitigate this issue, a derivative of DMAP covalently bound onto a solid silica support named SiliaBond can be used instead.^21,22^ This approach enables the recovery and potential recycling of the organic base, thereby reducing toxicity due to potential DMAP contamination of the end products. It is worth highlighting that the use of solid-supported reagents is increasing, owing to their significant potential to promote green chemical technologies. Thus, silica-supported catalysts have become widely adopted. They are easy to handle and reuse, considering the great stability of the silica material employed. Silica materials typically do not require preswelling, which makes their use far simpler and suitable for solid-state reactions. Therefore, our protocol was implemented with a solid-supported derivative of DMAP.

As shown in Scheme 1, all reactants (HA, DIC, and SiliaBond DMAP) were finely milled by a mortar and pestle, and then, the mixture was placed into a MW reaction vessel to be irradiated at 80 °C for 2 min. An excess (1.5 equiv) of SiliaBond DMAP relative to the amount of A9 peptide was employed. In fact, SiliaBond DMAP can activate the carboxylic function, thanks to its great nucleophilicity, by reacting with the O-acylisourea intermediate to form the acylated pyridinium ion, which promptly reacts with the amine group of the peptide in a subsequent step. This final amidation reaction was performed by adding A9 and a catalytic amount of K_2_CO_3_ to the activated HA, and then, the solid reactants were mechanically milled and transferred into the MW reactor to be irradiated under the same conditions (*T* = 80 °C for 2 min).

Next, the solid mixture was suspended in water, and the SiliaBond DMAP was easily removed by filtration. This purification method represents a great step forward, since the DMAP has a tendency to remain stuck on macromolecules such as polysaccharides.

### Structural Characterization

#### FT-IR Spectroscopy Characterization

The successful conjugation of A9 peptide to HA was first confirmed by infrared analysis. Characteristics peaks of HA (Figure 1) were identified at 3407 cm^–1^ (alcoholic OH stretching), 2930 cm^–1^ (C–H stretching), and 1624 cm^–1^ (C=O stretching of amide II).

**Figure 1:**
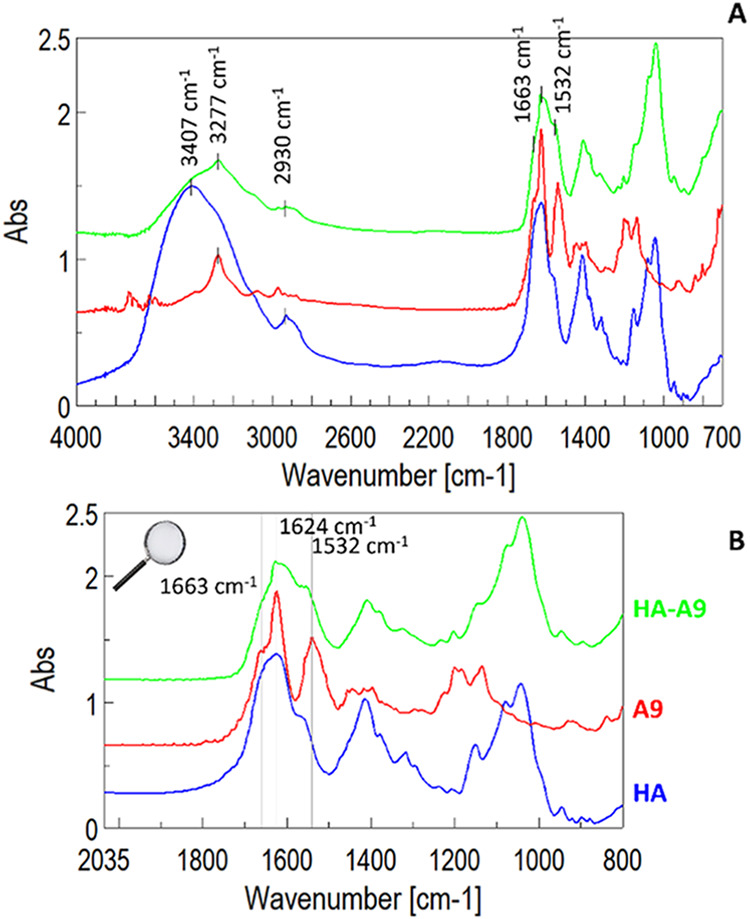
Normalized FT-IR spectra of HA-A9 conjugates (green), A9 (red), and HA (blue) (A); expansion of FT-IR spectra (B).

The chemical modification of HA produced changes in the FT-IR spectrum. New bands appeared at 3277 cm^–1^ (N–H amine II stretching), 1663 cm^–1^ (C=O amide II stretching), and 1532 cm^–1^(N–H amide II bending). Finally, the characteristic bands around 1000 cm^–1^ were typical of skeletal HA stretching. In Figure 1, representative spectra of the conjugate HA-A9, of native HA, and of the A9 peptide are reported.^23^

#### Nuclear Magnetic Resonance (^1^H NMR) Characterization

The NMR characterization of the A9-HA conjugate is quite challenging because the compound forms micelle-like aggregates (see below), which lead to substantial line-broadening and, ultimately, prevent NMR detection. To avoid the formation of micelles, the sample had to be dissolved at a very low concentration (<0.2 mg/mL), leading to ^1^H NMR spectra having a very poor signal-to-noise ratio. Notwithstanding such limitation, ^1^H NMR spectra in which signals from both the HA skeleton and the A9 peptide could be detected by accumulating a large number of scans (minimum 1024 scans). A representative spectrum of the HA-A9 conjugate is shown in the Supporting Information (Figure S1). The HA moiety of the conjugate shows two signals at 4.54 and 4.44 ppm (anomeric ring protons of the glucuronic acid and *N*-acetylglucosamine saccharide units), a set of resonances in the 4.0–3.0 ppm range (other sugar ring protons), a doublet at 8.05 ppm (*N*-acetylglucosamine amide proton), and a sharp singlet at 2.00 ppm (acetyl methyl group). Along with the signals of the carbohydrate backbone, the signals of the A9 peptide are clearly distinguishable. The assessment of the chemical structure of HA conjugates by NMR techniques is very often quite challenging because of solubility, conformational and structural heterogeneity, and aggregation issues.^20,24^ In our case, the very low sample concentration needed to avoid the formation of nanostructured aggregates (see below) hampered the acquisition of homo- or heterocorrelated 2D-NMR spectra, which are needed to confirm unambiguously the presence of the amide covalent bond between the peptide *N*-terminus and glucuronic acid carboxyl group.^20^ Assuming that A9 is linked through such a bond, the integral ratio between the acetyl methyl group of HA (singlet at 2.00 ppm) and the A9 valine methyl groups (two doublets around 0.81 ppm) could be used to calculate the degree of substitution (Figure 2).

**Figure 2:**
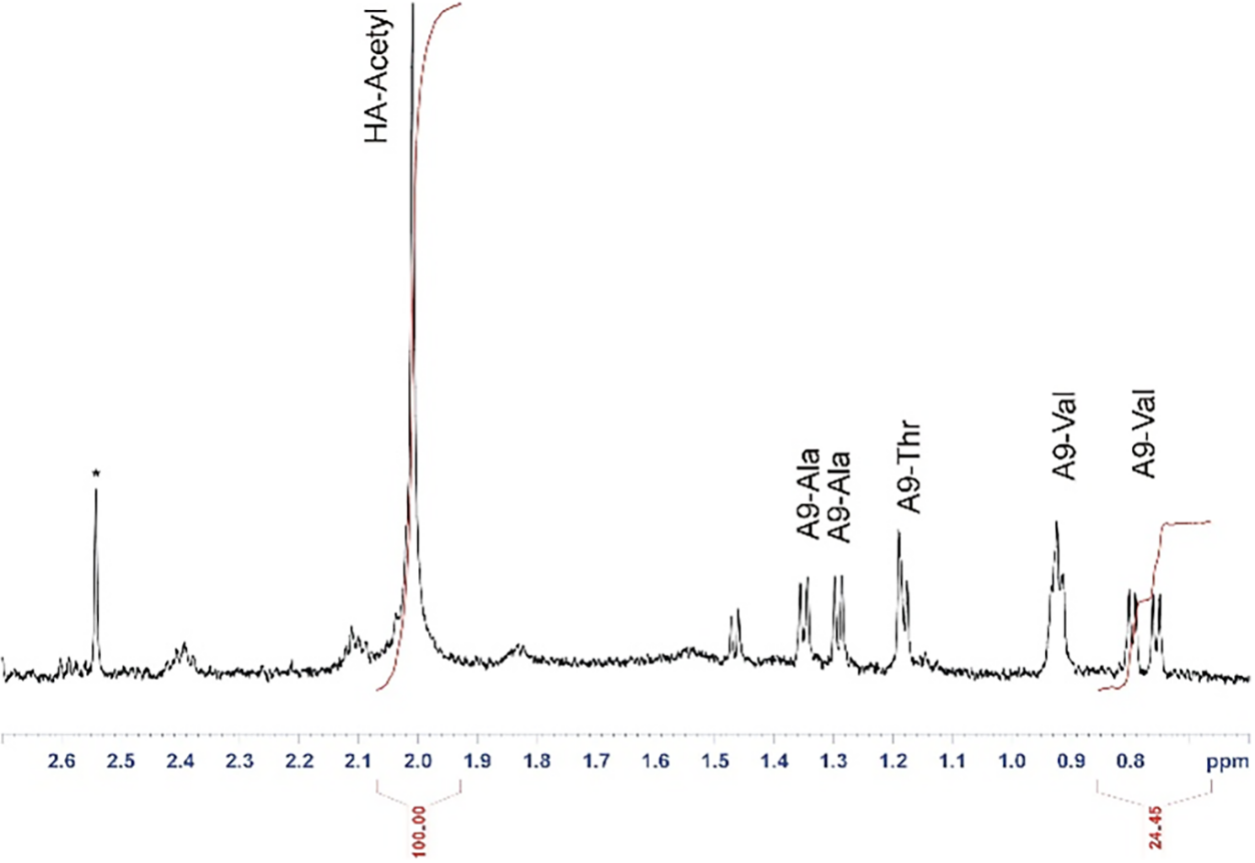
Aliphatic region of the 1H-NMR spectrum of A9-HA (600 MHz, H_2_O/D_2_O 600:50, 298 K, pH 4.5) with the integration of the acetyl signal of *N*-acetylglucosamine (3H) and of the methyl groups of one out of the two A9 valine residues (6H). The asterisks denote adventitious solvent impurity.

The DS was thus estimated in the range 0.02–0.12, depending on the reaction conditions, corresponding to a peptide mass content within the A9-HA conjugate in the range 6–24%m/m.

The successful covalent conjugation between the *N*-terminal amine group of the A9 peptide and the carboxylic function of HA was proven by comparison to a blank synthesis. The amidation reaction was carried out under the same experimental conditions as employed to obtain HA-A9, but without the addition of SiliaBond DMAP or DIC during the initial activation step. While in the absence of DMAP, an activation via symmetrical anhydride formation can only occur, in the absence of DIC, the carboxylic activation and the consequent amidation reaction cannot occur at all. We used the UV–vis absorbance peak of tryptophan at 280 nm to detect the presence of tryptophan, hence, the A9 peptide, in the final product. The characteristic absorption of the tryptophan chromophore belonging to the A9 peptide can be seen only in the products from the reaction carried out with all reagents (Figures 3 and S2–S4). Such an absorbance peak is not detectable in the absence of DIC and barely detectable in the absence of silica-supported DMAP. This indicates that the procedure leads to the formation of a covalent bond between HA and that the amount of A9 that could be potentially adsorbed by noncovalent interactions onto the polymer is very low.

**Figure 3:**
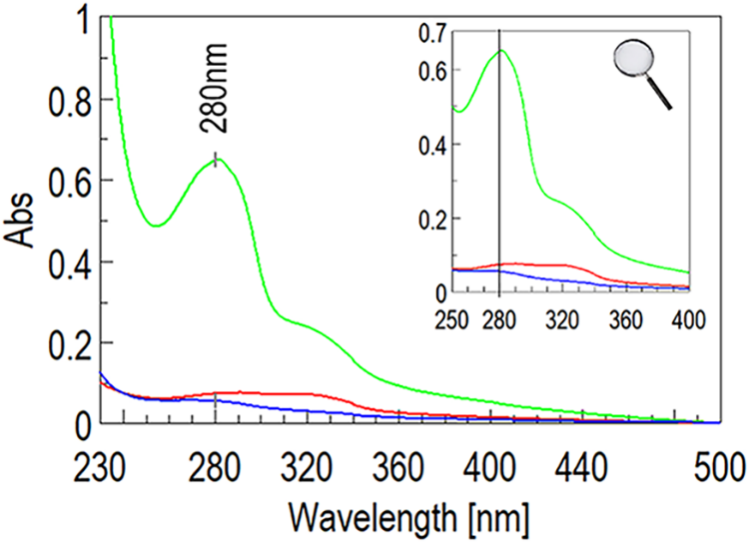
UV–vis spectra of HA-A9 obtained in different conditions: standard protocol with siliabond DMAP and DIC (light green), with DIC (blue), and with siliabond DMAP (red).

#### Evaluation of CMC and Investigation of HA-A9 Micelle Size Via Dynamic Light Scattering Technique

The self-assembling behavior of the synthesized amphiphilic conjugate was studied by means of the pyrene fluorescence method.^3^ By this approach, the solution was excited at 337 nm, and the emission spectrum was analyzed by evaluating the intensity ratio at 373 and 384 nm (*I*_1_/*I*_3_). The analysis of this ratio provides an indicator of the environment surrounding the pyrene molecules: a decrease in the *I*_1_/*I*_3_ ratio with an increase in the HA-A9 concentration suggests that pyrene is encapsulated within the hydrophobic interior of the aggregates formed by the polysaccharide conjugate.

The critical micellar concentration (CMC) was determined as the intersection of the two straight lines on the plot of the *I*_1_/*I*_3_ ratio versus HA–A9 concentration. The CMC values obtained fall within the range 0.10–0.20 mg/mL (∼0.115 mg/mL), indicating the concentration range where the amphiphilic conjugate begins to form aggregates (Figure 4).

**Figure 4:**
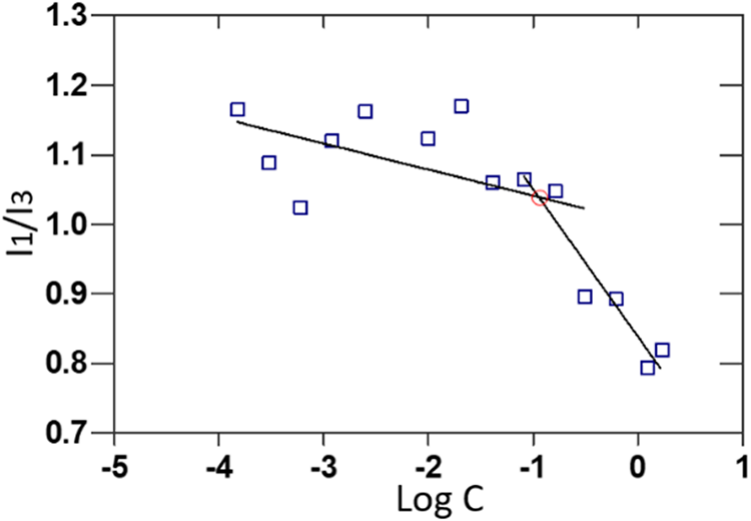
Determination of the CMC of HA-A9 conjugates.

Next, the nanoparticles of HA-A9 conjugate were prepared by dissolving an appropriate amount of the HA-A9 conjugate (>0.2 mg/mL) in 0.9% NaCl aqueous solution (see Materials and Methods) and sonicating for 30 min. The size distribution of the obtained aggregates was evaluated by the dynamic light scattering technique (DLS). The mean diameter of the HA-A9 derivative was in the range 304–443 nm (Figure 5 and Table 1).

**Figure 5:**
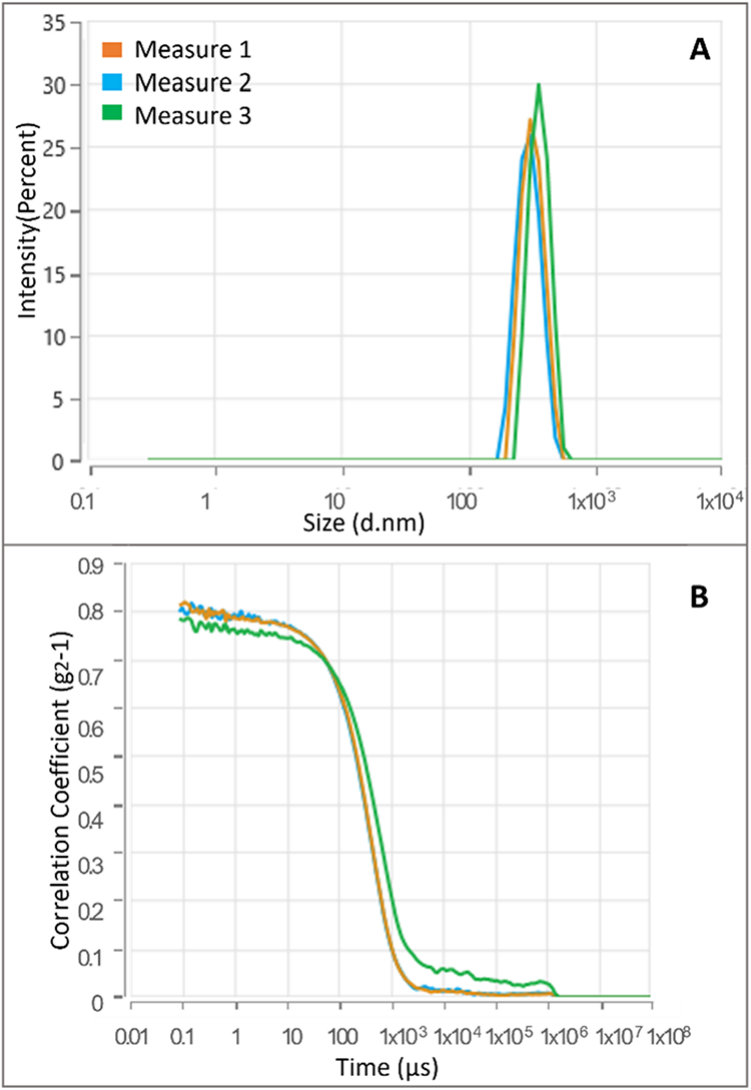
Size distribution (A) and intensity correlation functions of HA–A9 nanoparticles (B).

**Table 1:** Characteristic Parameters of the HA-A9 Nanoparticles

	mean diameter (nm)	PDI	δ (mV)
HA-A9	354 ± 77.36	0.217 ± 0.071	–21.7 ± 1.54

A narrow size distribution was found, as indicated by the polydispersity factor, which was always around 0.217. These results suggest that the developed HA-A9 conjugate has potential application as a micellar system capable of delivering anticancer agents to tumor cells through the specific interaction of the A9 ligand with the HER2 receptor, which is overexpressed on the cell surface.

#### Fluorescence Binding Investigation

The binding affinity of the HA-A9 conjugate for its receptor model, HER2-DIVMP, was evaluated by using the fluorescence spectroscopy titration method already described to characterize bimolecular complexes.^14–16^

Briefly, with such methodology, both tyrosine and tryptophan are excited at a wavelength of 280 nm, while only the tryptophanyl fluorescence is monitored at 354 nm. As already reported for the A9/HER2-DIVMP bimolecular complex, tyrosine residue *Y*_568_ of the receptor could come sufficiently close to the tryptophan residue of A9, thereby enhancing its fluorescence emission at 354 nm. Consequently, changes in fluorescence emission at 354 nm can be considered as indicators of both binding affinity and binding topology. The plot of the fluorescence intensity difference at 354 nm (Δ*F*_354_), obtained by subtracting the separate contributions of the two tryptophan (*W*_35_ of the peptide ligand and *W*_592_ of the receptor fragment) as a function of increasing concentrations of the A9 ligand, either in the presence or absence of a fixed concentration of the HER2-DIVMP receptor, gives a hyperbola-shaped binding isotherm from which the binding affinity constant *K*_d_ can be calculated.

Titrations were carried out with the HA-A9 conjugate (λ_ex = 280 nm) at receptor concentrations between 0.05 and 0.15 μM. These consistently caused a change in fluorescence at 354 nm, which was positive, as expected. A typical plot of Δ*F*_354 versus ligand concentration is shown in Figure 6.

**Figure 6:**
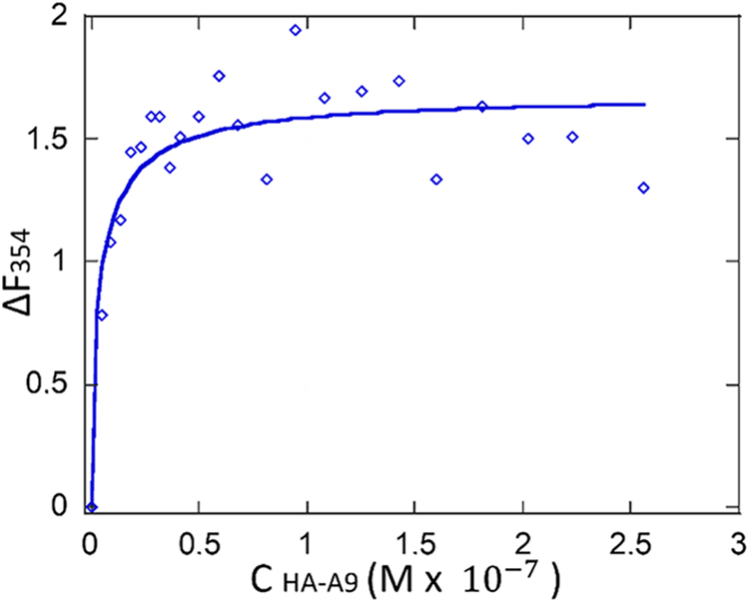
Standard titration binding curve of HA-A9 to HER2-DIVMP (10 mM phosphate buffer, pH = 7.2). The employed receptor concentration was 0.15 μM. The values on the ordinate are the residual fluorescence signals at 354 nm, obtained upon subtraction of each contribution from HER2-DIVMP and the peptide ligand. The amount of ligand is expressed in terms of concentration of the A9 peptide (*C*_HA‑A9_).

The functional shape and the sign of the HA-A9 titration curve were similar to those obtained with the monomeric A9 peptide. This suggested that both the multimeric and monomeric forms of A9 might share the same binding topology with the HER2-DIVMP receptor model.

Using computer-assisted fitting of the binding curves, based on a model for saturable specific interaction, we found a dissociation constant (*K*_d_) of 6.01 ± 1.5 nM. This is lower than the value found for the A9 monomer (10.3 ± 3.6 nM), which indicates that the HA-A9 multimer is characterized by an increased binding affinity (Figure 6).

#### Circular Dichroism Characterization

Self-assembling peptides bound to a polymeric scaffold, such as a natural polysaccharide, can lead to a three-dimensional hydrogel organization, in which peptide aggregates often are composed of β-sheet structures. These high-water-content networks have the potential to encapsulate and deliver therapeutics as well as to be useful biomimetic materials.

Based on these considerations, the structure of HA-A9 was analyzed using circular dichroism to assess the β-sheet content of the peptide portion of the conjugate. Initial measurements were conducted at various pH values, but no significant differences in the structure could be detected. Then, the CD analysis was performed at different concentrations of A9 peptide (0.026 and 0.13 mM) within the HA-A9 conjugate. As shown in Figure 7, an increase in the β-sheet structure could be detected from the CD analysis.

**Figure 7:**
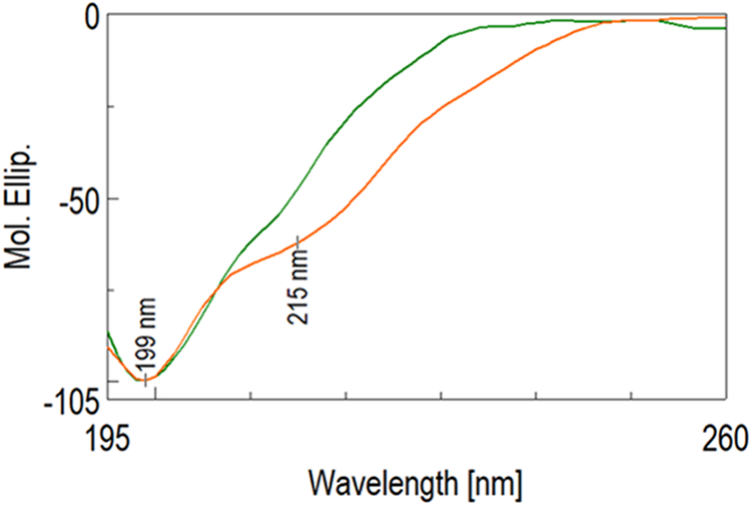
Normalized CD spectra at different concentrations: 0.026 mM (dark green) and 0.13 mM (orange) of HA-A9 in water at 20 °C.

This secondary structure content is evidenced by the negative band centered at around 215 nm. The results obtained by the structural analysis indicate the potential of forming novel HA-based hydrogel to be employed in the biomedical field.^25–28^

## Conclusion

A novel, green procedure to conjugate a peptide to a natural polysaccharide was developed. An amidation reaction was performed under solvent-free conditions and consisted of a solid-state mechanically assisted conjugation. The reaction was promoted by microwave radiation as an energy supplier. The activation of the carboxylic groups present on the polysaccharide was successfully performed by using a commercially available organo-catalyst Si-DMAP supported on solid silica. The possibility to eliminate the organic base by filtration along with the possibility to reuse the solid-supported reagent makes the conjugation strategy even more sustainable in terms of reuse and chemical consumption. So far, Si-DMAP has been successfully employed in liquid media for batch catalysis, as well as in a continuous flow setup.^21^ To the best of our knowledge, the commercial Siliabond material—specifically DMAP immobilized on silica gel, has demonstrated, for the first time, notable efficiency in facilitating a reaction carried out entirely at the solid-state.

The new synthetic approach has been applied to obtain the multimerization of the A9 ligand by linking multiple copies of the ligand on a natural polysaccharide. The obtained multicopy system of A9 demonstrated an increased binding affinity toward the HER2-DIVMP receptor model, compared to the binding properties of the A9 peptide alone. This was evaluated by using fluorescence spectroscopic methods.

In addition, the HA-A9 conjugate proved to be able to self-assemble into micelle-like structures and also, at higher concentration, to have the propensity to form viscous material, characterized by increased content of β-sheet peptide structure. Both supramolecular structural organizations are potentially suitable for applications in targeted drug delivery or as biocompatible materials.

## Supplementary Material



## Notes

1H NMR spectra of dimer HA-A9; UV–vis spectra of HA-A9 performed using standard protocol with siliabond DMAP and DIC, DIC, and siliabond DMAP alone (PDF)

## Abbreviations

1H-NMR: proton nuclear magnetic resonance
2D-NMR: two-dimensional nuclear magnetic resonance spectroscopy
2D-NOESY: two-dimensional nuclear overhauser effect spectroscopy
A9: H_2_N–QDVNTAVAW–CONH_2_
CD: circular dichroism
CH_3_CN: acetonitrile
CMC: critical micellar concentration
D2O: deuterium oxide
DIC: *N*,*N*′-diisopropylcarbodiimide
DIPEA: diisopropylethylamine
DMAP: 4-(dimethylamino)­pyridine
DLS: dynamic light scattering
ECM: extra-cellular matrix
FT-IR: Fourier transform infrared spectroscopy
HA: hyaluronic acid
HER2: human epidermal growth factor receptor 2
HER2-DIVMP: human epidermal growth factor receptor 2
HOBt: *N*-hydroxybenzotriazole
K_2_CO_3_: potassium carbonate
*K*_d_: dissociation constant
LC-ESI-MS: liquid chromatography-electrospray ionization-mass spectrometry
MW: microwave radiation
NMR spectroscopy: nuclear magnetic resonance spectroscopy
PEG: polyethylene glycol
PyBOP: benzotriazol-1-yl-oxy-tris-pyrrolidino-phosphonium
RP-HPLC: reversed-phase high-performance liquid chromatography
Si-DMAP: SiliaBond DMAP
TFA: trifluoroacetic acid
TIS: triisopropyl silane
UV–vis: ultraviolet visible
W: tryptophan
Y: tyrosine
