# Supplementary material for: Green Synthesis of a Hyaluronan-Based Multimeric A9 Peptide System: Polysaccharide-A9 Conjugate with Enhanced HER2 Receptor Binding Affinity and Potential Biomedical Applications as an Active Material
Source: Biomacromolecules. 2025 Nov 18;26(12):8622–9. doi: 10.1021/acs.biomac.5c01553 (PMC12690571; doi:10.1021/acs.biomac.5c01553)
Supplement: Supplementary file 1 [file bm5c01553_si_001.pdf]

SUPPORTING INFORMATION

Green Synthesis of a Hyaluronan-Based Multimeric A9 peptide System: polysaccharide-A9 conjugate with enhanced HER2 receptor binding affinity and potential biomedical applications as active material

Valentina Verdoliva,[1, †] Alfio Pulvirenti,[2, †] Giuseppe Digilio,[3] Stefania De Luca[2, *]

[1] Department of Environmental, Biological and Pharmaceutical Sciences and Technologies, National Research Council (CNR), Institute of Crystallography, Via Vivaldi, 43, 81100 Caserta, Italy.

[2]Department of Biomedical Sciences, Institute of Biostructures and Bioimaging, National Research Council (CNR), Via P. Castellino, 111, 80131 Naples, Italy.

[3]Department of Science and Technological Innovation, Università del Piemonte Orientale "A. Avogadro", 15121 Alessandria, Italy.

† These authors equally contributed to this work.

Corresponding author's email address: stefania.deluca@cnr.it

Institute of Biostructures and Bioimaging, National Research Council (CNR), 80131 Naples, Italy;

orcid.org/0000-0002-7078-1696.

TABLE OF CONTENTS

- **^{1}H-NMR spectra of HA-A9……………………………………………...S2**
- **UV-vis spectrua of HA-A9 performed using siliabond DMAP and DIC, siliabond DMAP, DIC………………………………………………………………………………….S3**

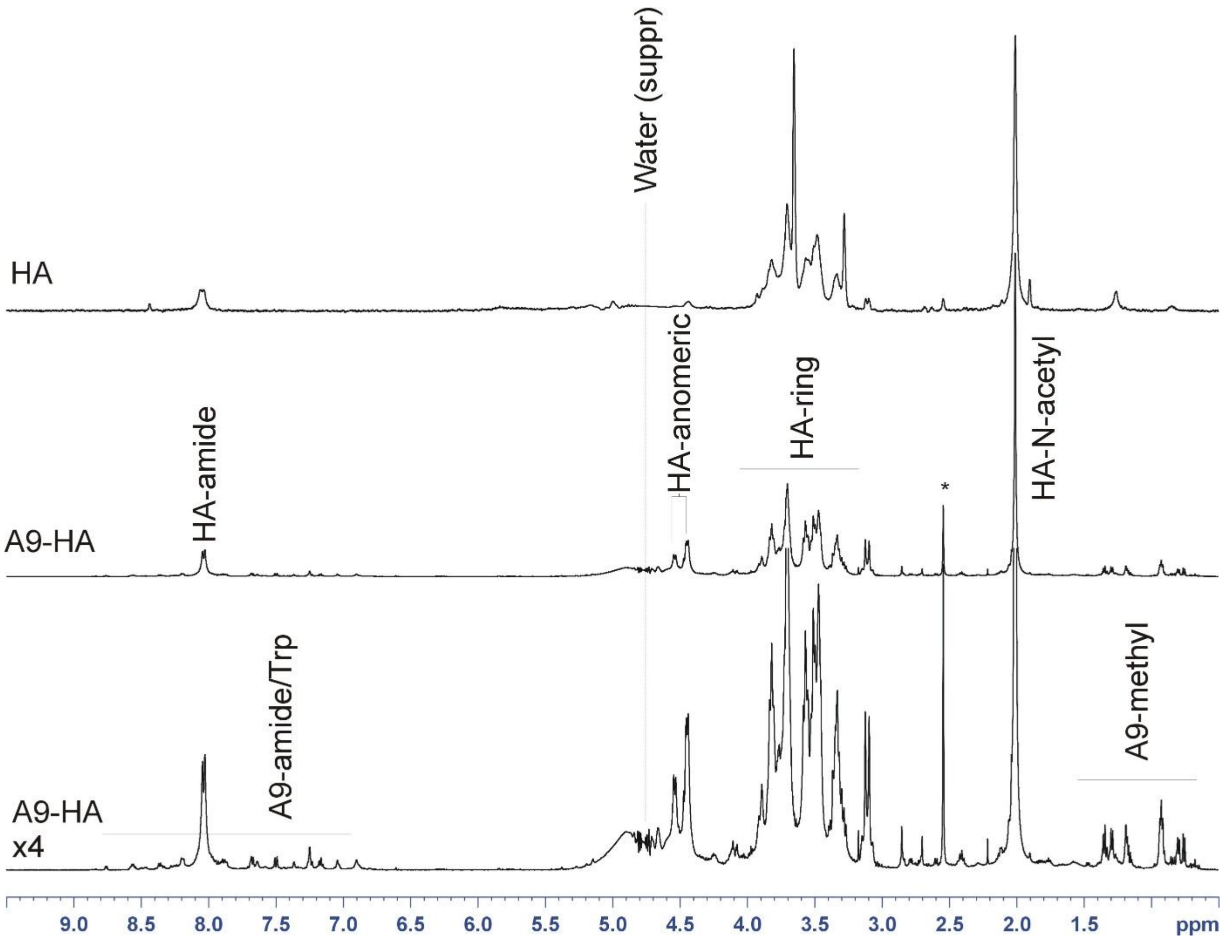

Figure S1A. Top: Full view of the ^{1}H-NMR spectrum of hyaluronic acid before conjugation with A9 (400 MHz, H_2O/D_2O 550:50, 298 K, pH 5). Middle: Full view of the ^{1}H-NMR spectrum of the A9-HA conjugate (batch-2, 600 MHz, H_2O/D_2O 550:50, 298 K, pH 5). Bottom: Y-expansion of the same ^{1}H-NMR spectrum shown in the middle. All spectra were acquired in H_2O/D_2O 550:50 mixture with suppression of the very intense water signal by means of the excitation sculpting scheme (see experimental) to allow for the detection of HA and A9 peptide amide signals. The dotted line marks the position of the suppressed water signal. The asterisk denotes an adventitious solvent impurity.

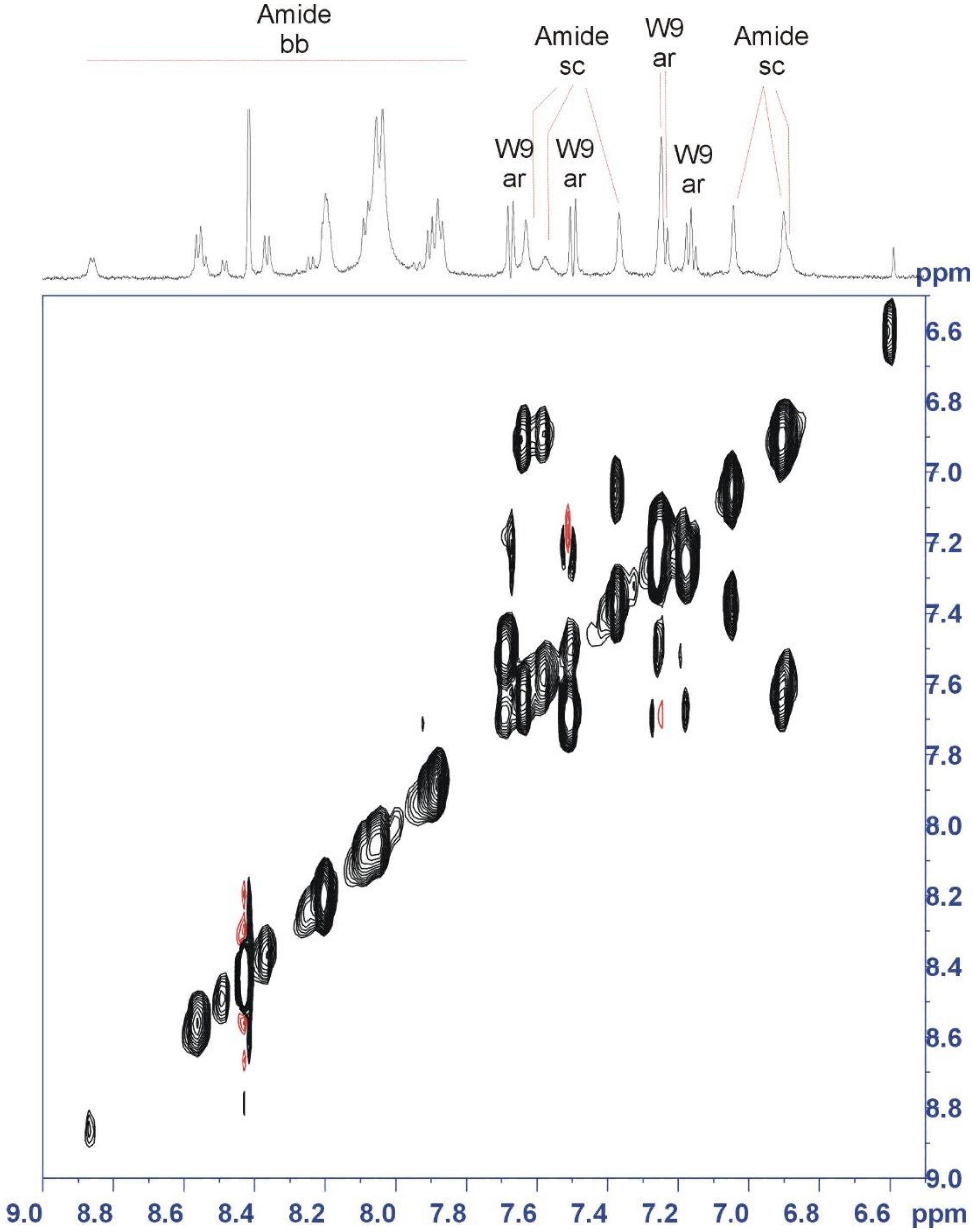

Figure S1B. 2D-TOCSY NMR spectrum of a9-HA (batch-3, mixing time 100 ms, 600 MHz, H_2O/D_2O 550:50, 298 K, pH 5). The spectrum was acquired by means of the Bruker pulse program mlevesgpph pulse program with water suppression by excitation sculpting and gradient pulses. Acquisition parameters included mixing time 100 ms, time domain 2048 x 256, number of scans 300, relaxation delay 2.5 s. The expansion of the amide/aromatic region is shown with partial assignment (*Amide Sc*: side chain or C-terminus amide proton signals; *W9 ar*: signals form the Trp indole ring; *Amide bb*: backbone amide protons; *Amide HA*: N-acetyl glucosamine amide signal of HA).

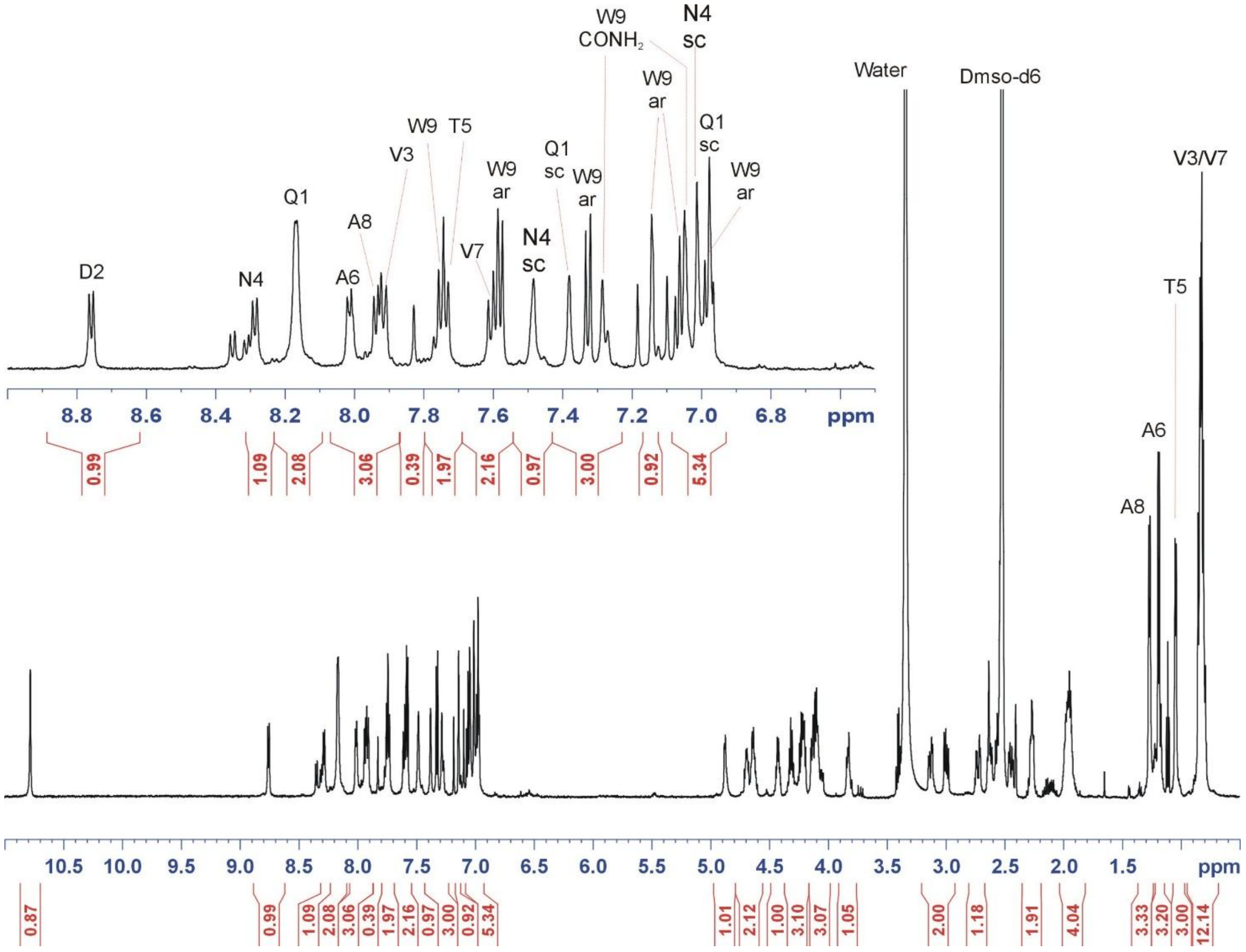

Figure S1C. ^{1}H-NMR reference spectrum of the A9 peptide (600 MHz, dmso-d_6, 298K), with assignment of the resonances in the amide, aromatic and methyl regions. For further details about the assignment, see Verdoliva *et al. J. Med. Chem.* **2025**, 68, 16299-16305.

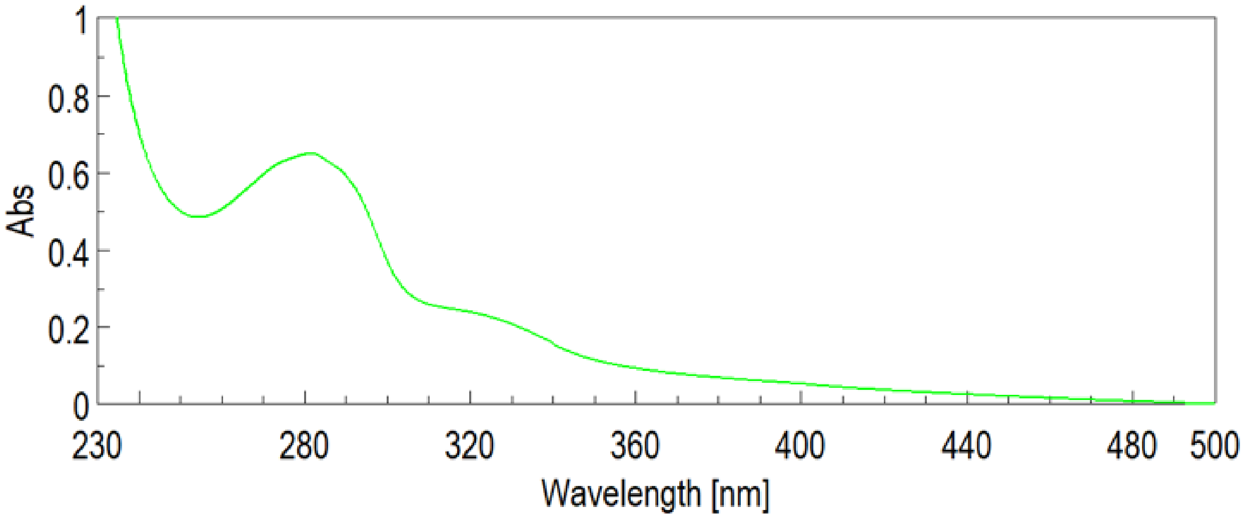

Figure S2. UV-vis spectrum of HA-A9 performed using siliabond DMAP and DIC

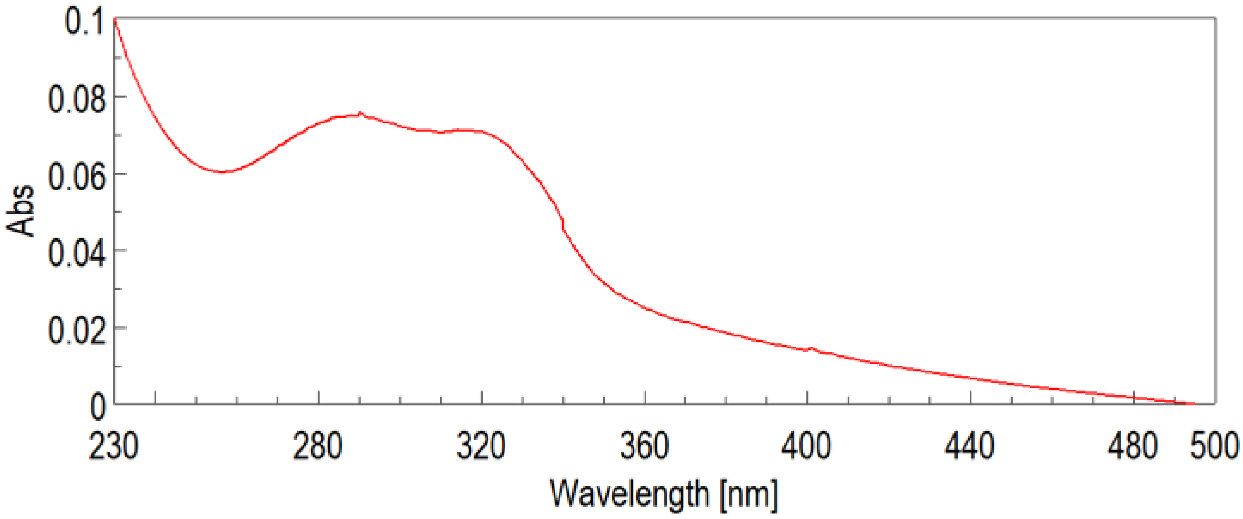

Figure S3. UV-vis spectrum of HA-A9 performed using siliabond DMAP

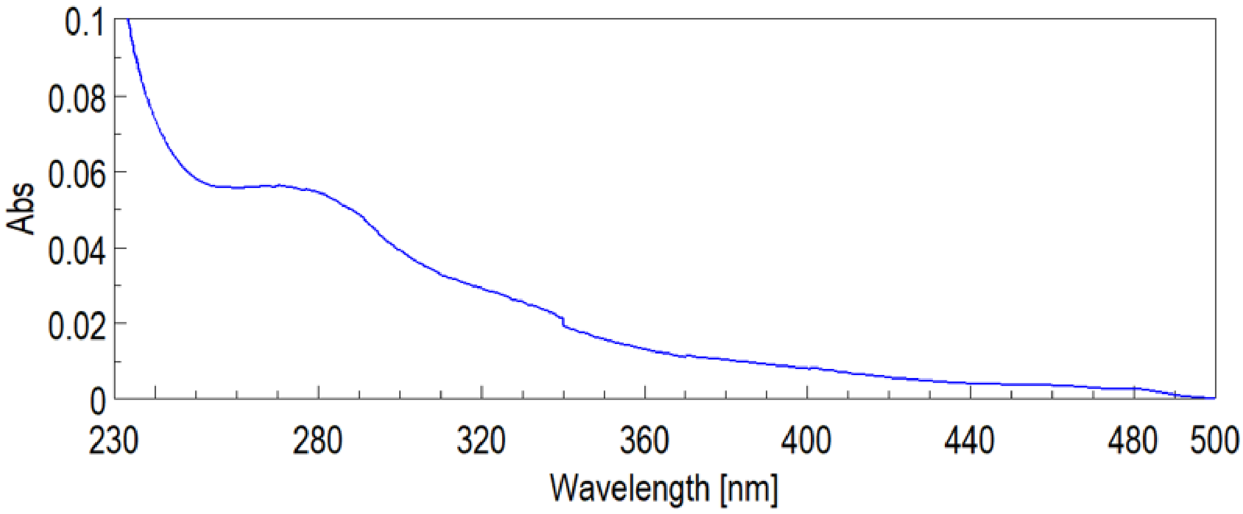

Figure S4. UV-vis spectrum of HA-A9 performed using siliabond DIC
